# Palatal bone thickness for mini-implant insertion in different vertical growth patterns: a systematic review

**DOI:** 10.21142/2523-2754-1102-2023-152

**Published:** 2023-06-29

**Authors:** Nguyen Puente de la Vega Mendigure, Denisse Rosario Bashualdo Candia, Valery Valer Jáuregui

**Affiliations:** Division of orthodontic, Universidad Científica del Sur. Lima, Peru. puentevm3@gmail.com, dnic_b@hotmail.com, valeryvvj@gmail.com Universidad Científica del Sur Division of orthodontic Universidad Científica del Sur Lima Peru puentevm3@gmail.com dnic_b@hotmail.com valeryvvj@gmail.com

**Keywords:** palatal bone thickness, orthodontic anchorage device, vertical growth pattern, grosor del hueso palatino, dispositivo de anclaje ortodóncico, patrón de crecimiento vertical

## Abstract

**Introduction:**

The purpose of this systematic review was to identify, evaluate, and provide information about palatal bone thickness in different vertical growth patterns for the placement of orthodontic anchorage devices.

**Methods:**

We performed a systematic review of the published data in Medline via PubMed, Web of Science, Cochrane Library, and Scopus from January 2000 to August 2022 using eligibility criteria. Data collection analysis and data extraction were performed independently by three reviewers. Sensitivity analyses were performed with the Cochrane risk of bias tool and the ROBINS-I tool was used for non-randomized studies.

**Results:**

A total of 343 articles were identified. The inclusion criteria included palatal bone thickness and vertical facial growth. However, both variables were found in 4 studies and only 2 had a control group. The different studies evaluated palatal bone thickness according to sex (male 14.1 mm; female 9.68 mm) and vertical malocclusion (normal 2.2 -12.6 mm; open bite 1.9 -13.2mm) with heterogeneous results. Likewise, the vertical growth pattern with a low angle (9.39 mm) was greater than the normal (8.55 mm) and high angle (7.53 mm).

**Conclusions:**

Palatal bone thickness varies according tp different vertical growth patterns, with the greatest thickness being found near the incisive foramen in hypodivergent individuals.

## RESUMEN

**Introducción:**

El propósito de esta revisión sistemática fue identificar, evaluar y brindar información sobre el espesor óseo palatino en diferentes patrones de crecimiento vertical para la colocación de dispositivos de anclaje ortodónticos**.**

**Materiales y métodos:**

Realizamos una revisión sistemática de los datos publicados de Medline, a través de PubMed, Web of Science, Cochrane Library y Scopus. Se realizaron búsquedas sistemáticas con criterios de elegibilidad desde enero de 2000 hasta agosto de 2022. Tres revisores realizaron de forma independiente el análisis de recopilación de datos y la extracción de datos. Los análisis de sensibilidad se realizaron con la herramienta Cochrane de riesgo de sesgo y para estudios no aleatorios seleccionados de intervenciones se utilizó la herramienta ROBINS-I.

**Resultados:**

Se identificaron un total de 343 artículos. La elegibilidad de los estudios incluyó el grosor del hueso palatino y el crecimiento facial vertical. Sin embargo, ambas variables se encontraron en 4 estudios y solo 2 tenían grupo control. El espesor óseo palatino presentó resultados heterogéneos, los estudios evaluaron el espesor óseo palatino en cuanto al sexo (masculino: 14,1 mm; femenino: 9,68 mm), y maloclusión vertical (normal: 2,2-12,6 mm; mordida abierta: 1,9-13,2 mm). Asimismo, el patrón de crecimiento vertical con ángulo bajo (9,39 mm) fue mayor que el ángulo normal (8,55 mm) y ángulo alto (7,53 mm).

**Conclusiones:**

El grosor del hueso palatino muestra diferencia en los patrones de crecimiento vertical, el mayor grosor se encontró cerca del foramen incisivo en individuos hipodivergentes.

## INTRODUCTION

Palatal bone thickness is determined by the ossification of both sides of maxillary extensions during facial growth and development. This thickness of the skeletal structure may differ according to vertical growth patterns ^1^^,^^2^. In orthodontic treatments, the palatal bone surface serves as the insertion site for temporary anchorage devices (TADs) to control the biomechanical effects of orthodontic treatment, reduce treatment time, and thereby benefit both patients and clinicians ^3^

The thickness of the palate is the distance from the superior cortical (nasal floor) to the inferior cortical (hard palate). The ideal TAD insertion zone is clinically delimited between the projection of the contact point between the upper canines and the first premolar to mesial of the first molar, in the middle and lateral part of the palatal vault ^1^^,^^4^.

TADs used in the bone palate reduce the risk of inflammation and injury because the micro screws are placed beyond the intrinsic anatomical structures, and also increase the possibility of different types of biomechanics for orthodontic treatment ^5^^,^^6^. The keratinized tissue of the palate has advantages for the placement of TADs, such as a large amount of fibro mucosal tissue, easy access for insertion, low risk of blood vessel injury and, in some cases, the presence of these devices on the palatal surface is not perceived. Failure of TAD insertion is related to the thickness and density of bone tissue. Therefore, the risk of failure is diminished when a TAD is placed in the palatal bone ^7^^,^^8^.

Cone-beam computed tomography (CBCT) imaging provides sagittal, transverse, and vertical planes of the palate bone as well as accurate, quantifiable, and reproducible 3D images of this zone ^3^^,^^9^.

Facial growth types are classified as hypodivergent, norm divergent, and hyperdivergent according to the cephalometric criteria of Bjork ^10^. Likewise, Ricketts ^11^ also described facial biotypes, such as brachyfacial, mesofacial and dolichofacial, characterized by different vertical and transverse variations patterns ^7^. A specific skeletal facial biotype presents specific maxillary bone features such as the shape and bone dimensions. Moreover, this growth and development show variations that differentiate each biotype ^10^^,^^11^. Likewise, the hard palate is also influenced by changes in facial growth and development that consequently influence the bone type, which is important for TAD placement. In addition, it is necessary to quantify the thickness of the palatal bone for the placement of TADs, considering possible differences in cases with vertical malocclusions that may present greater or lesser bone thickness. Therefore, the purpose of this systematic review was to compare palatal bone thickness for mini-implant insertion in different vertical facial growth patterns.

## MATERIAL AND METHODS

### Protocol and registration

This article followed the PRISMA guide for systematic reviews, fulfilling the 27 items included. We also recorded this systematic review in PROSPERO with the registration number PROSPERO 2020 CRD42020176187 Available from: https://www.crd.york.ac.uk/prospero/display_record.php?ID=CRD42020176187

### Eligibility criteria

The eligibility criteria included articles that evaluate the thickness of the palatal bone with different vertical facial growth patterns using observational designs, cases and controls, cohorts, randomized and non-randomized clinical trials published since January 2000 (Table 1).

**Table 1:** Eligibility criteria used for study selection.

Category	Inclusion criteria	Exclusion criteria
Participants	Individuals with different vertical growth patterns that need orthodontic treatment with a mini-implant	Nonhuman studies (animal or laboratory studies)
Intervention	Individuals with vertical growth pattern (hypodivergent, and hyperdivergent)	Patients or sides treated with any other techniques or drugs for tooth movement acceleration
Control	Norm divergent individuals	
Outcome	Palatal bone thickness.	

### Information sources

Electronic searches were conducted in Medline via PubMed, Web of Science, Cochrane Library, Scopus from January 2000 to 2021. There was no language or year or publication status restriction for inclusion.

### Search strategy

The information from the scientific searches was carried out by identifying the concept in the same PubMed database in Mesh language. Word variations were searched in the Cochrane database using an advanced search. The detailed strategy in all sources is presented in Table 2.

**Table 2:** Search strategy in the different databases

Data base	Electronic database Set of terms (key terms)
Medline vía PubMed	((Vertical facial pattern or vertical growth or vertical malocclusion or vertical facial type or hypodivergent or hyperdivergent or vertical facial growth pattern or vertical skeletal pattern) AND (quantitative or assessment or palatal bone or palatal or hard palatal or Palatal bone thickness or palatal thickness or palatine bone thickness)) AND (Orthodontic anchorage procedure or temporary anchorage device or skeletal anchorage procedure or TADs or miniscrew or mini implant or microscrew or mini dental implant or mini-implant or miniimplant or micro-screw or skeletal Anchorage)
Scopus	(Vertical AND facial AND pattern OR vertical AND growth OR vertical AND malocclusion OR vertical AND facial AND type OR hypodivergent OR hyperdivergent OR vertical AND facial AND growth AND pattern OR vertical AND skeletal AND pattern) AND (orthodontic AND anchorage AND procedure OR temporary AND anchorage AND device OR skeletal AND anchorage AND procedure OR tads OR miniscrew OR mini AND implant OR microscrew OR mini AND dental AND implant OR mini-implant OR miniimplant OR micro-screw OR skeletal AND anchorage) AND ( "Palatal bone thickness" OR "palatal thickness" OR palate OR palatine AND bone OR "palatine bone thickness")
Cochrane Library	((Vertical facial pattern or vertical growth or vertical malocclusion or vertical facial type or hypodivergent or hyperdivergent or vertical facial growth pattern or vertical skeletal pattern) AND (quantitative or palatal bone or palatal or hard palatal or Palatal bone thickness or palatal thickness or palatine bone thickness)) AND (Orthodontic anchorage procedure or temporary anchorage device or skeletal anchorage procedure or TADs or miniscrew or mini implant or microscrew or mini dental implant or mini-implant or miniimplant or micro-screw or skeletal Anchorage) in Title Abstract Keyword - (Word variations have been searched)
Web of Science	#1 TS=(Vertical facial pattern OR vertical growth OR vertical malocclusion) #2 TS=(Quantitative or Palatal bone thickness or palatal thickness or palate or palatine bone or palatine bone thickness or hard palate) #3 TS=(Orthodontic anchorage procedure or temporary anchorage device or skeletal anchorage procedure or TADs or miniscrew or mini implant or microscrew or mini dental implant or mini-implant or miniimplant or micro-screw or skeletal Anchorage) #3 AND #2 AND #1

### Study selection

The selection of the studies consisted of 2 phases.

During the first phase, two authors (NPVM, DRBC) independently evaluated the titles and/or abstracts. References that met the eligibility criteria were included. Full reference texts with insufficient information in the title and/or abstract for a decision on inclusion or exclusion were retrieved for evaluation in phase 2.

During the second phase of article selection, the same authors independently evaluated the full texts, including studies that met the eligibility criteria.

In both phases, differences were resolved by consensus. If necessary, a third author (VVJV) assessed whether or not the study was included. For the second phase, the reasons for exclusion from the review were also recorded.

### Data extraction and items extracted

- Two reviewers (NPVM, DRBC) independently screened the titles and abstracts identified from the electronic databases and additional sources and duplicates were removed. Next, full articles were retrieved to confirm their eligibility based on the inclusion criteria. The reviewers independently selected the articles for inclusion in the qualitative synthesis. Disagreements were resolved by verbal discussion and clarifications from the reviewers and consultation with a third reviewer (VVJV), when necessary.
- The following data were independently extracted by two reviewers using an excel spreadsheet: Autor year, Study design, sex and age, Descriptions of group, Characteristics and intervention protocol and Result (palatal bone thickness). If necessary, the author was contacted by email to clarify any doubts about the study.

### Assessment of bias risk within studies

The risk of bias assessment of the non-randomized prospective and retrospective studies was performed with Risk of Bias in Non-randomized Studies of interventions (ROBINS-I Tool) of the Cochrane Collaboration. Seven domains were considered: bias due to confounding; bias in the selection of study participants; bias in the classification of interventions; bias due to deviations from intended interventions; bias due to missing data, bias in the measurement of outcomes; and bias in the selection of the reported results.

The review independently assessed the risk of bias of the included studies and discrepancies were solved by verbal discussion and consultation with a third reviewer (VVJV), if necessary. 

The level of evidence was assessed using the Robins-I tool of the Cochrane Collaboration available online at https://www.riskofbias.info/welcome/home/current-version-of-robins-i, for evaluating the number of studies included, the study designs, risk of bias, inconsistency, indirectness, imprecision, and other considerations (such as publication bias). The tool uses seven domains for determining the overall bias. Depending on the seriousness of the limitation in each of these domains, the evidence may be downgraded by five levels. Based on this assessment, the certainty of the evaluation of the outcome could be: low, moderate, serious, critical and no information of bias. The level of evidence was assessed separately for observational studies.

### Summary measurements

Measurements for the primary outcome were based on continuous data (millimeters in CBCT), but measurements for the secondary outcome were based on different anatomic references. Table 3 shows the study characteristics and the results of the studies included. 

**Table 3:** Risk of bias assessment of selected studies

	Pre - Intervention		At intervention		Post - Intervention				
	Bias due to confounding	Bias in selection of participants into the study	Bias in classification of interventions	Bias due to deviations from intended interventions	Bias due to missing data	Bias in measurement of outcomes	Bias in selection of the reported result	Overall
Suteerapongpun. et al., 2018	Low	Low	low	Low	low	moderate	low	moderate
Wang et al., 2017	Low	Low	moderate	Low	moderate	low	low	moderate
Jayakumar et al., 2012	low	Moderate	moderate	Low	low	low	low	moderate
Gracco et al., 2008	Low	Low	low	Low	low	moderate	low	moderate

ROBINS-I, Risk of Bias in Non-randomized Studies of Interventions.

### Synthesis of results

The data collected was ordered with the outcome variable. Relative properties of homogeneity or heterogeneity were sought with the outcome of palatal bone thickness or vertical growth.

The results of the studies selected were heterogeneous and therefore a meta-analysis was not performed.

### Risk of bias of across studies

Risk of bias assessment was carried out with the indicators proposed by the Robbins I tool (non-randomized articles) to determine if the methodologies proposed by the studies had internal validity and if the results were reliable to allow comparison with other studies with the same variables (Table 4).

**Table 4:** Summary of study characteristics and results of the studies included

Reference	No (male:female)	Mean age (ys)	Age range	Method	Sites	Interval ML (mm)	Interval AP (mm)	Software	Reference line	Site of max VBH (AP: ML)	VBH (mm)
Suteerapongpun. et al., 2018	8:22	Not stated	15-30	CBCT	From the middle of the distal bony margin of the incisor foramen, laterally from the mid-sagittal plane (both sides)	0,3,6,9,12	3,6,9,12, 15,18,21, 24	Planmeca Romexis Viewer 2.3.1.R (Planmeca Oy, Helsinki, Finlandia)	Incisor foramen and SNP	Normal bite: 9:12 Open Bite: 13:7	9.12
Wang et al., 2017	57:66	26,8	18-40	CBCT	28 (left side)	0, 3, 6, 9	0,4,8,12, 16,20,24	KaVo 3D eXam (KavoSybron, Orange, CA)	Incisor foramen PNS	Low-angle: 9.39 Normal-angle: 8.55 High-angle: 7.53	9.39
Jayakumar et al., 2012	30:30	18,5	Grupo A: 15-24 Grupo B: 25-35	TC	15 (right side)	0, 3, 6	4, 8, 12, 24,28	software medical CAD, (Imita)	Incisor foramen and lower border of incisor foramen	Male: 14.1 Female: 9.68	14.1
Gracco et al., 2008	80:82	Not stated	10-44	CBCT	20 (both sides)	-	4, 8, 16, 24	Newtom 3G software	Incisor foramen	AP:10.3	10.3

Ys : years; ML: mesio lateral; AP: Antero posterior; PNS: posterior nasal spine; SNP: single nucleotide polymorphism

## RESULT

### Study selection

**Figure 1:**
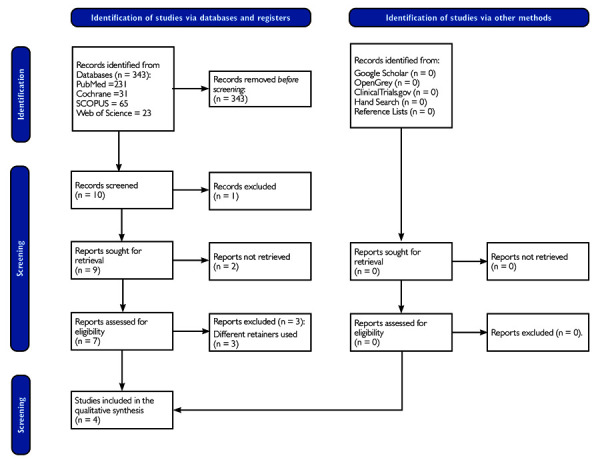
Flow diagram of the selection of studies included in the present review

A total of 343 studies were initially identified through the database search, with 2 studies being excluded due to systematic review design. The flow diagram for the selection of studies is shown in Figure 1. The selection included 231 studies found in Medline via PubMed, 23 studies in the Web of Science digital database, 31 from the Cochrane Library, and 65 studies in Scopus. 

### Study characteristics

Table 2 shows the characteristics of the 4 studies included in this systematic review. Only 2 study were retrospectives observational studies with a control group, while the other 2 studies were retrospective observational without a control group but had co-variables such as sex and age. 

Of the 343 studies screened, 4 studies met the inclusion criteria and duplicated studies were excluded. Among the remaining 4 studies, 3 used three-dimensional CBCT while 1 used CT for measuring bone height and 1 for bone density (Table 3). 

The different studies presented heterogeneous results in palatal bone thickness, with some investigating palatal bone thickness and sex (male 14.1 mm; female 9.68 mm), while also evaluated differences in vertical malocclusion (normal 2.2 -12.6 mm; open bite 1.9 -13.2), also in the medial lateral position (8.7mm), and in the anteroposterior position (10.3 mm). 

## DISCUSSION

### Evaluation and insertion of TADs

In the scientific literature review Björk.^10^ described different palatal bone thicknesses according to facial vertical growth patterns. However, despite these differences the palatal bone was adequate for TAD placement. Other studies described that the stability and longevity of TADs in orthodontic treatment was influenced by palatal cortical thickness,^9^^,^^13^ palatal bone thickness,^14^^-^^17^ vertical growth patterns,^7^ and sex.^16^ Likewise, one study suggested that muscular activity on the bone tissues and the pressure of the tongue can modify, albeit not significantly, palatal bone thickness.^14^ Age is an important factor in the process of growth and development of patients, with CBCTs of adult patients showing an increased thickness of the palatal bone cortex, being more adequate for the placement of TADs.

Multiple studies have reported that the thickness of the cortical bone can have a strong impact on the primary stability essential for successful TAD placement. The elements of orthodontic biomechanics include tooth movement and local anatomy, both of which define the decision of where to place a TAD.18 These individual variations may be present within the local anatomy and thus, three-dimensional images are necessary to obtain quantitative information.^19^^,^^20^

The thickness of the palatal bone is the most accepted site for TAD insertion due to the lower incidence of inflammatory processes and because it is surrounded by fibrous palatal gingiva. The objective of TAD insertion allows good control of orthodontic biomechanics and ensures satisfactory results.^9^ Some authors, such as Poorsattar-Bejeh et al.^4^, Ryu et al.^20^, and Gracco et al.^15^, agree that the surface of the palate is a suitable area for the insertion of mini-screws. Likewise, the thickness of the palatal bone is influenced by gender, with male patients presenting greater palatal bone thickness compared to females.^17^

### Identification of vertical malocclusion

In the CBCT study of Suteerapongpun et al.^19^ evaluating vertical growth, they found that the thickness of the palatal bone was significantly greater in cases with a normal than in an open bite, mainly due to the incisor and the mediolateral foramen in the palatal suture.

Furthermore, in CBCTs of individuals with vertical growth, Wang et al.^7^ observed that a low angle presents a greater palatal bone thickness compared to a normal and high angle, with these anatomical characteristics being commonly observed in orthodontics and when choosing the type of anchorage to use. The angle of growth is inversely related to the median and posterior thickness of the palate.

In general, the inclusion of only 4 studies in the present systematic review, does not allow definitive conclusions to be made on the subject and more well-controlled research with good methodological structure should be carried out. Therefore, the results of this review cannot be generalized, although it seems that the thickness of the palatal bone differs according to the patterns of vertical growth, with the greatest thickness being found near the incisive foramen in hypodivergent individuals.

## CONCLUSIONS

The thickness of the palatal bone differs according to the vertical growth patterns, with the greatest thickness being found near the incisive foramen in hypodivergent individuals.
